# Phenotypic and Genotypic Analysis of Newly Obtained Interspecific Hybrids in the *Campanula* Genus

**DOI:** 10.1371/journal.pone.0137537

**Published:** 2015-09-09

**Authors:** Anna-Catharina Röper, Jihad Orabi, Henrik Lütken, Brian Christensen, Anne-Marie Thonning Skou, Renate Müller

**Affiliations:** 1 Department of Plant and Environmental Sciences, Faculty of Science, University of Copenhagen, Højbakkegaard Allé 9-13, 2630, Taastrup, Denmark; 2 Department of Plant and Environmental Science, Faculty of Science, University of Copenhagen, Thorvaldsensvej 40, 1871, Frederiksberg, Denmark; 3 AgroTech A/S, Institute for Agri-Technology and Food Innovation, Højbakkegaard Allé 21, 2630, Taastrup, Denmark; Università Politecnica delle Marche, ITALY

## Abstract

Interspecific hybridisation creates new phenotypes within several ornamental plant species including the *Campanula* genus. We have employed phenotypic and genotypic methods to analyse and evaluate interspecific hybridisation among cultivars of four *Campanula* species, i.e. *C*. *cochleariifolia*, C. *isophylla*, *C*. *medium* and *C*. *formanekiana*. Hybrids were analysed using amplified fragment length polymorphism (AFLP), flow cytometry and biometrical measurements. Results of correlation matrices demonstrated heterogeneous phenotypes for the parental species, which confirmed our basic premise for new phenotypes of interspecific hybrids. AFLP assays confirmed the hybridity and identified self-pollinated plants. Limitation of flow cytometry analysis detection was observed while detecting the hybridity status of two closely related parents, e.g. *C*. *cochleariiafolia* × *C*. *isophylla*. Phenotypic characteristics such as shoot habitus and flower colour were strongly influenced by one of the parental species in most crosses. Rooting analysis revealed that inferior rooting quality occurred more often in interspecific hybrids than in the parental species. Only interspecific hybrid lines of *C*. *formanekiana* ‘White’ × *C*. *medium* ‘Pink’ showed a high rooting level. Phenotype analyses demonstrated a separation from the interspecific hybrid lines of *C*. *formanekiana* ‘White’ × *C*. *medium* ‘Pink’ to the other clustered hybrids of *C*. *formanekiana* and *C*. *medium*. In our study we demonstrated that the use of correlation matrices is a suitable tool for identifying suitable cross material. This study presents a comprehensive overview for analysing newly obtained interspecific hybrids. The chosen methods can be used as guidance for analyses for further interspecific hybrids in *Campanula*, as well as in other ornamental species.

## Introduction

With more than 300 species, *Campanula* is one of the largest genera within the Campanulaceae family [1]. Many *Campanula* species distributed in the Mediterranean and Balkan regions have been classified thoroughly and their phylogeny has been determined [2–4]. The inflorescences which comprise one or more flowers, with a tubular corolla which is funnel formed or rotated, are characteristic for this ornamental genus. The corolla colour is mostly blue or dark, seldom white [1]. Analysis from Roquet et al. proved that the *Campanula* core is divided into two main groups: the *C*. *rapunculus* L. clade and the *Campanula s*. *str*. clade [4]. The genetic diversity within several cultivated plant genera has been diminished due to the breeding focusing on few traits. Commercially important *Campanula* cultivars are derived from few species e.g. *C*. *formanekiana*, *C*. *medium*, *C*. *isophylla and C*. *portenschlagiana*, by which *C*. *portenschlagiana* is the most produced species with 19 mio. potted plants in Denmark in 2013 [5].

Wide hybridisation between genera and species is a tool to increase genetic variability by introgression of new traits. Interspecific hybridisation is often prevented due to hybridisation barriers. These are caused either by prezygotic through gametic incompatibilities or by postzygotic barriers through failed endosperm development [6,7]. The greater the genetic distance, the lower the chance of achieving a successful hybridisation. Model plants such as *Arabidopsis thaliana* have been investigated intensely to identify barriers that inhibit interspecific hybridisation [8–10]. In *A*. *thaliana*, barriers due to ploidy level and to the epigenetic status of donor and recipient genomes were detected [9,10]. Additionally, commercially important ornamental plants need to be investigated to verify whether mechanisms identified in model plants are universal in different genetic backgrounds. Interspecific hybrids were also obtained from several ornamental interspecific hybrids e.g. *Jatropha*, *Helleborus* and *Cyclamen* [11–13].

During recent years understanding of the molecular structure of interspecific crossing barriers has increased. Failed development of the endosperm plays a key role in obtaining hybrids of parental species with different ploidy levels, due to an imbalance of imprinted genes [14]. In *A*. *thaliana*, the role of imprinted genes and parent-of-origin gene expression due to differences in DNA methylation of ovule and pollen were related to the endosperm development [9,15]. Delay in flower induction as phenotypic expression for hybridisation incompatibilities was also explored in *A*. *thaliana* [16]. Another indicator for incompatibility in wide hybridisation is albinism [17]. Albinism occurs when both nuclear and chloroplast genomes are incompatible. Whether the plastid DNA (ptDNA) has been inherited maternally, paternally or biparentally, it is always species specific and was explored in diverse interspecific crosses of *Azalea* [17]. One indicator for unidirectional inheritance is when only one cross direction results in albino plants as observed for interspecific hybridisation in *Lonicera caerulea* × *L*. *gracilipes* [18].

For assessing genetic diversity among the germplasm of plant species, different DNA based marker methods have been constructed e.g. restriction fragment length polymorphisms (RFLP) [19], random amplified polymorphic DNA (RAPD) [20] or amplified fragment-length polymorphism (AFLP) [21]. AFLP was chosen as a method in this study because no prior knowledge of the DNA sequence is needed. Furthermore, the results are reproducible and reliable [21]. In *Campanula*, AFLP has been applied to distinguish among different species from the *C*. *rapunculus* clade and the *Campanula s*.*str*. clade [22,23].

In a previous study, interspecific hybrids between cultivars of *C*. *medium* (*Cm*) and *C*. *formanekiana* (*Cf*) were produced by ovule culture [24]. Additional hybrid lines were obtained by the breeder PKM A/S, Odense, Denmark. In the present study interspecific hybrids from both sources were analysed. Ten interspecific hybrids in total were selected for detailed morphological and molecular investigations. Biometrical data of important breeding traits were analysed and hybridity was proven. Moreover, genetic distances between parental species and offspring were determined by DNA molecular markers. Two methods, flow cytometry and AFLP were used to identify interspecific hybrids in the *Campanula* genus.

The overall aim of the present study was to investigate the genetic influence of the parental species on the phenotype of obtained interspecific hybrids.

## Materials and Methods

### Plant Material, Cultivation and Experimental Design

In total ten interspecific *Campanula* hybrids and four parental *Campanula* species were used for morphological characterisation and molecular proof of hybridity (Table 1). All species originate from South-Eastern Europe, have the same ploidy level (2n) and 32 or 34 chromosomes [25–30] (Table 2). For each interspecific cross combination two hybrid lines were selected. The plant material was produced by the breeder PKM A/S (Odense, Denmark). When possible ten cuttings were taken for each interspecific hybrid and parental plant species (Table 1) and placed in 11 cm pots with soil type Special recipe 1 (Pindstrup, Ryomgaard, Denmark), containing 0–20 mm peat size with 15% perlite (pH 5.4–6), cultivated at 19°C with a photoperiod of 9 h and a photosynthetic photon flux density (PPFD) of 80–140 μmol m^2^ s^-1^ (Lucalox 1U, Gavita, Andebu, Norway).

**Table 1:** Parental species and interspecific hybrids, with period of morphological analyses and number of plants used.

		Maternal plant			Paternal plant				
Cross type*	Genotype code	species r	cultiva		species	cultivar	Abbreviation of species and Hybrids	Morphological analyses	Number of cuttings for morphological analyses
P	A	*C*. *medium*	Pink ‘Sweet MEE’				*Cm*P	August- October 2013	8
P	B	*C*. *medium*	*Dark* ‘Sweet MEE’				*Cm*D	August- October 2013	10
P	C	*C*. *formanekiana*	White ‘Mary MEE’				*Cf*W	January- March 2014	10
P	D	*C*. *formanekiana*	Blue ‘Mary MEE’				*Cf*B	January- March 2014	10
S	E1	*C*. *medium*	Pink ‘Sweet MEE’	×	*C*. *medium*	Pink ‘Sweet MEE’	*Cm*P *× Cm*P	August- October 2013	8
S	E2	*C*. *medium*	Pink ‘Sweet MEE’	×	*C*. *medium*	Pink ‘Sweet MEE’	*Cm*P *× Cm*P	August- October 2013	8
I	F1	*C*. *medium*	Pink ‘Sweet MEE’	×	*C*. *formanekiana*	Blue ‘Mary MEE’	*Cm*P *x Cf*B	August- October 2013	10
I	F2	*C*. *medium*	Pink ‘Sweet MEE’	×	*C*. *formanekiana*	Blue ‘Mary MEE’	*Cm*P *x Cf*B	August- October 2013	10
I	G1	*C*. *formanekiana*	Blue ‘Mary MEE’	×	*C*. *medium*	Pink ‘Sweet MEE’	*Cf*B *x Cm*P	January- March 2014	9
I	G2	*C*. *formanekiana*	Blue ‘Mary MEE’	×	*C*. *medium*	Pink ‘Sweet MEE’	*Cf*B *x Cm*P	January- March 2014	9
I	H1	*C*. *formanekiana*	White ‘Mary MEE’	×	*C*. *medium*	*Dark* ‘Sweet MEE’	*Cf*W *x Cm*D	January- March 2014	4
I	H2	*C*. *formanekiana*	White ‘Mary MEE’	×	*C*. *medium*	*Dark* ‘Sweet MEE’	*Cf*W *x Cm*D	January- March 2014	8
I	I1	*C*. *formanekiana*	White ‘Mary MEE’	×	*C*. *medium*	Pink ‘Sweet MEE’	*Cf*W *x Cm*P	January- March 2014	9
I	I2	*C*. *formanekiana*	White ‘Mary MEE’	×	*C*. *medium*	Pink ‘Sweet MEE’	*Cf*W *x Cm*P	January- March 2014	7
P	J	*C*. *isophylla*	‘Starina’				*Ci*	September–October 2013	1
P	K	*C*. *cochleariifolia*	n.a.				*Cc*	September–October 2013	1
I	L1	*C*. *cochleariifolia*	n.a.	×	*C*. *isophylla*	‘Starina’	*Cc x Ci*	September-October 2013	8
I	L2	*C*. *cochleariifolia*	n.a.	×	*C*. *isophylla*	‘Starina’	*Cc x Ci*	September-October 2013	10

*P = parental species; I = Interspecific hybrid; S = self-pollination

**Table 2:** Origin, ploidy and chromosome number from selected *Campanula* species.

*Campanula* species	Place of origin	Chromosome number and ploidy
*C*. *cochleariifolia*	European Alps	34 (2n)
*C*. *formanekiana*	Greece and Macedonia	34 (2n)
*C*. *isophylla*	North-Western Italy	32 (2n)
*C*. *medium*	Southern Eastern Europe, Balkan	34 (2n)
*C*. *poscharskayana*	Serbia, Montenegro, Bosnia and Herzegovina, Croatia	34 (2n)
*C*. *portenschlagiana*	Serbia, Montenegro, Bosnia and Herzegovina, Croatia	34 (2n)

[25–30]

After ten weeks the plants were vernalised at 5°C to induce flowering. When *C*. *formanekiana* was used as a maternal plant, the interspecific hybrids required six weeks of vernalisation. *C*. *medium* cultivars did not need vernalisation to induce flowers, but in order to give all genotypes the same treatment, all *C*. *medium* cultivars and interspecific hybrids, when *C*. *medium* was used as maternal plant, were exposed to a vernalisation of three weeks. All genotypes were acclimatised for seven weeks at 18°C, 18 h photoperiod supplemented with photosynthetic photon flux density (PPFD) of 100 μmol m^2^ s^-1^ (Lucalox 1U, Gavita A/S, Andebu, Norway). After acclimatisation the plant material was transported to The University of Copenhagen (Taastrup, Denmark) for conducting the morphological characterisation at 20°C, 18 h of photoperiod supplemented with PPFD of approximately 165 μmol m^2^ s^-1^ (MASTER SON-T PIA Hg Free 400W/ E E40, Philips, Amsterdam, The Netherlands). The *Campanula* cultivars are mentioned throughout the text by abbreviations. The full names are shown in Table 1.

The plant material was divided into two parallel experiments, placed separately in different parts of the greenhouse, each containing five plants of each interspecific hybrid and the parental plant species. The experiments were carried out as a randomized block design with five blocks containing one plant in each block. As the selected parental plant species and their progeny exhibited different periods for flower induction, the biometrical data were collected during the following 3 periods: August to October 2013, September to October 2013 and January to March 2014 (Table 1).

### Morphological Characterisation

For the characterisation of the obtained interspecific hybrids, 13 biometric parameters were selected and measured. The first open and wilted flower was recorded three times per week. Flowering time (FT) was defined as the period from the opening of the first flower to the first wilted flower. The first and the second open flower (OF) were labelled and two days later flower diameter and length (FD, FL) were measured. At the time point of the first open flower the greenness of the leaves was evaluated by measuring the relative chlorophyll content (CC) and Chlorophyll Content Index (CCI) using a chlorophyll content meter (Chlorophyll content meter, CCM-200 plus, Apogee Instruments, Logan, UT, USA). CCI values around 1 describe leaves with nearly no chlorophyll content, i.e. an albino plant. The root formation (RF) was scored in three categories 1, 2 and 3, whereby root levels 1 and 3 exhibit the lowest and highest root formation level, respectively.

When the first wilted flower was monitored, pollen quality (P) was analysed by staining the pollen with 1% (w/v) acetocarmine (1 g carmine powder dissolved in 45 ml glacial acetic acid and 55 ml ddH_2_O) [31]. For this purpose, two open flowers were harvested and the pollen was removed and placed on a glass slide with three drops of 1% (w/v) acetocarmine. After 10 min it was possible to identify red stained pollen grains as potentially fertile pollen by using a light microscope (DM750, Leica, Wetzlar, Germany) [31]. When possible, a minimum of 100 pollen grains were examined from each sample. Furthermore, the number of open flowers per plant (NFP), total plant height and diameter of the plant (PH, PD) were recorded. Finally, the fresh and dry weight (FW, DW) (after 72 h at 70°C) was determined. All results are averages with standard error.

### Chromosome Counting

Fresh, white root tips of approx. 2 cm in length were collected and fixed in α- monobromnaphtalene solution (6 ml dist. H_2_0 with 2 drops of 1- monobromnaphtalene) (Sigma B73104, St. Louis, MO, USA) for 4 h. Afterwards, the roots were transferred into a Clark solution of (1:3) acetic acid glacial (Scharlau AC0344, Barcelona, Spain) and 99% ethanol (VWR, Darmstadt, Germany) and kept for 24 hours at room temperature. Roots were then stored at -20°C for 48 h, and then the solution was changed to 70% ethanol. Root tips remained then at -20°C until slide preparation. Rinsed root tips were placed twice in 0.01 M citrate buffer (citric acid pH 4.6, (Honeywell, Seelze, Germany) with tri-sodium-citrat dihydrat (Honeywell, Seelze, Germany) under gentle orbital shaking (Rotamax 120, Heidolph, Schwabach, Germany) for 10–15 min. Root tips were then transferred to an enzyme solution (20% (v/v) pectinase (*Aspergillus niger*, Sigma 17389, St. Louis, MO, USA) and 2% (w/v) cellulase (R-10 C8001, Saveen Werner, Limhamn, Sweden) for 2 min at 37°C. Roots were again placed in new citrate buffer for a minimum of 15 minutes under gentle orbital shaking. Afterwards, a root tip of approximately 1 cm was cut with a sharp scalpel by using a stereo microscope (Tagarno, TM 320, Horsens, Denmark) and placed on a microscopy slide with a drop of 45% aqueous acetic acid for 3 minutes. Root tips were completely chopped to release the inner cells and placed on a slide with a cover slip. The slide was warmed up under a flame and the cover slip was tapped to remove air bubbles, warmed again and then gently pressed with the thumb. After that the slide was exposed to liquid nitrogen for approximately 10 seconds and the cover slip was removed gently. Finally, 2 μl/ml DAPI staining solution (PanReac AppliChem A4900, Darmstadt, Germany) was added and dry samples were fixed with 27 μl mounting medium CITIFLUOR (Citifluor Ltd, London, Great Britain). The examination was followed using a fluorescence microscope (Leica DM 2000 and fluorescence source Leica EL 6000, Solms, Germany).

### Flow Cytometry Detection of Relative DNA Content

For analysis of the relative DNA content each sample was treated according to the protocol of Partec CyStain UV Precise P (Partec, Münster, Germany) [32]. For each sample, 0.5 cm^2^ of leaf material was chopped with a razor blade for one minute and incubated in 400 μl extraction buffer for five minutes (Partec, Münster, Germany). Afterwards, the liquid solution was poured through a 30 μm CellTrics Disposable Filter (Partec, Münster, Germany). This was followed by the addition of 1.6 ml DAPI staining solution (4.6 diamidino-2-phenyldole) nuclei staining buffer (Partec, Münster, Germany) to the sample, which was then incubated in darkness for a minimum of one minute. Finally, the fluorescence of the nuclei was measured by the flow cytometer BD FACSAria III U (Becton Dickinson Biosciences, Franklin Lakes, NJ, USA). Fluorescence was excited by a 405 nm laser and DAPI detector from 430 to 470 nm counting in total 10,000 events. The threshold for DAPI-H was set to 20,000. Mean fluorescence intensity, standard deviation of the mean (SD), coefficient of variation (CV) and counts of stained nuclei were calculated based on the two replicates with the software Flowjo V10 (www.flowjo.com).

### DNA Extraction and AFLP

Three to four leaves (approx. 12 cm^2^) per plant were collected and freeze dried (Table 1). DNA was isolated using the CTAB protocol [33]. AFLP assay was carried out as described by Vos et al (1995), with the following modifications: digestion was performed in a total reaction volume of 10 μl by adding 500 ng genomic DNA, 0.5 M NaCl, 1 x T4 ligase-buffer with ATP DNA, 1 U of *Mse*I and 2 U of *Pst*l. The digestion was performed at 37°C for 90 min followed by 65°C for 90 min. The ligation mix containing 1 x T4 DNA ligase-buffer with ATP, 25 μM *Mse1*, 2.5 μM *Pstl* and 2 U/μl T4 DNA ligase (Fermentas, Thermo Scientific, Slangerup, Denmark) was added to the digestion reaction. After incubation for 3 hours at 37°C, all samples were diluted 10 fold with ddH_2_O.

Pre-amplification was performed by adding 1x Extra buffer (15 mM MgCl_2_), 4 mM dNTP-Mix and pre-selective Pstl and Msel primers and 5 U/μl Taq-DNA-polymerase together. Finally, 16 μl of the pre-amplification mix and 4 μl of each restriction/ligation reaction were mixed. The pre-amplification PCR product was diluted 10 fold by adding ddH_2_O. Main amplification was conducted in 1x Extra buffer (15 mmM MgCl_2_), 10 mM MgCl_2_, 4 mM dNTP-Mix, 5 μM Msel selective primer, 1 μM Pstl selective primer and 5 U/μl Taq-DNA-Polymerase. To each 17 μl of the master mix 3 μl of the diluted pre-amplification product was used as a template. Four AFLP primer combinations were selected based on the number of informative bands to amplify the DNA (S1 Fig): M50/ P16, M62/P20, M47/ P35 and M49/ P11 (http://wheat.pw.usda.gov/ggpages/keygeneAFLPs.html accessed 09.05.14). Pstl primers were labelled with different fluorescent dyes.

### Detection and Genotyping

AFLP fragments were detected using the AB 3130xl Genetic Analyzer (Applied Biosystems, Foster City, CA, USA). Analysis of DNA fragments and genotyping was carried out using GeneMarker version 2.5.2 (Softgenetics LLC, State College, PA, USA). Polymorphic bands with a size ranging from 52 to 581 base pairs were scored, either as band present (1) or absent (0). Samples showing an unusual curve pattern were excluded from further analysis. The result was a binary matrix including 309 loci. Genetic information is given in S1 Table.

### Data Analysis

Calculation for correlation matrix and principal component analysis (PCA) was based on selected biometrical parameters of plant height (PH), plant diameter (PD), fresh weight (FW), dry weight (DW), pollen quality (PQ) and flowering time (FT). For presenting the heterogeneity of the used species for interspecific hybridisation, the selected biometrical parameters were analysed using a correlation matrix ([34,35]). The correlations between the parameters were displayed in a correlation matrix by giving Pearson correlation coefficients. The correlation coefficient represents positive correlation of maximum 1 (both parameters increase together) and a maximum negative correlation of -1 (if one parameter increases the other decreases). If no correlation exits between the parameters, the correlation coefficient is expressed as 0. Correlations were calculated separately for each species. P-values were calculated and presented in the S1 Table. The principal component analysis (PCA) allowed for interpreting the relationships between parental species and interspecific hybrids based on 13 biometrical parameters.

Genetic distances based on the AFLP data were estimated using Jaccard’s dissimilarity index. Jaccard’s dissimilarity index was calculated as follows:

$$J^{\prime }=\frac{{\text{M}}_{01}+{\text{M}}_{10}}{{\text{M}}_{01}+{\text{M}}_{10}+{\text{M}}_{11}}$$

where M_01_ represents the total number of markers, assuming that accession *i* presents no band (0), while accession *j* does present a band (1); M_10_ represents the total number of markers, where accession *i* presents a band (1), and accession *j* is 0; and M_11_ represents the total number of markers, assuming that both *i* and *j* present a band (i.e., double presence of the same allele). Cases in which both *i* and *j* are (0) were ignored, as such a scenario cannot be confirmed due to the dominant nature of the AFLP markers. Distance-based Neighbour-Net-unrooted trees were created using SplitsTree4 [36] (version 4.13.1). The Bootstrap method was applied to check confidentiality.

All analysis of variance and multiple comparisons of means of the following morphological parameters: NFP, quotient of FD and FL and the CCI, were analysed by Tukey Contrast tests using R software, whereby an error of 5% was accepted. Additionally, p-values were Holm adjusted to take inflated false positives due to multiple comparisons into account. Descriptive statistics of PCA were conducted with the R package ‘FactoMineR’ [37]. Correlation matrices were created utilising the R package ‘PerformanceAnalytics’ [38]. The average Mean, SD, Median and CV were calculated from the values of relative DNA content and analysed using the software Flowjo V10.

## Results

### Breeding Material

**Fig 1:**
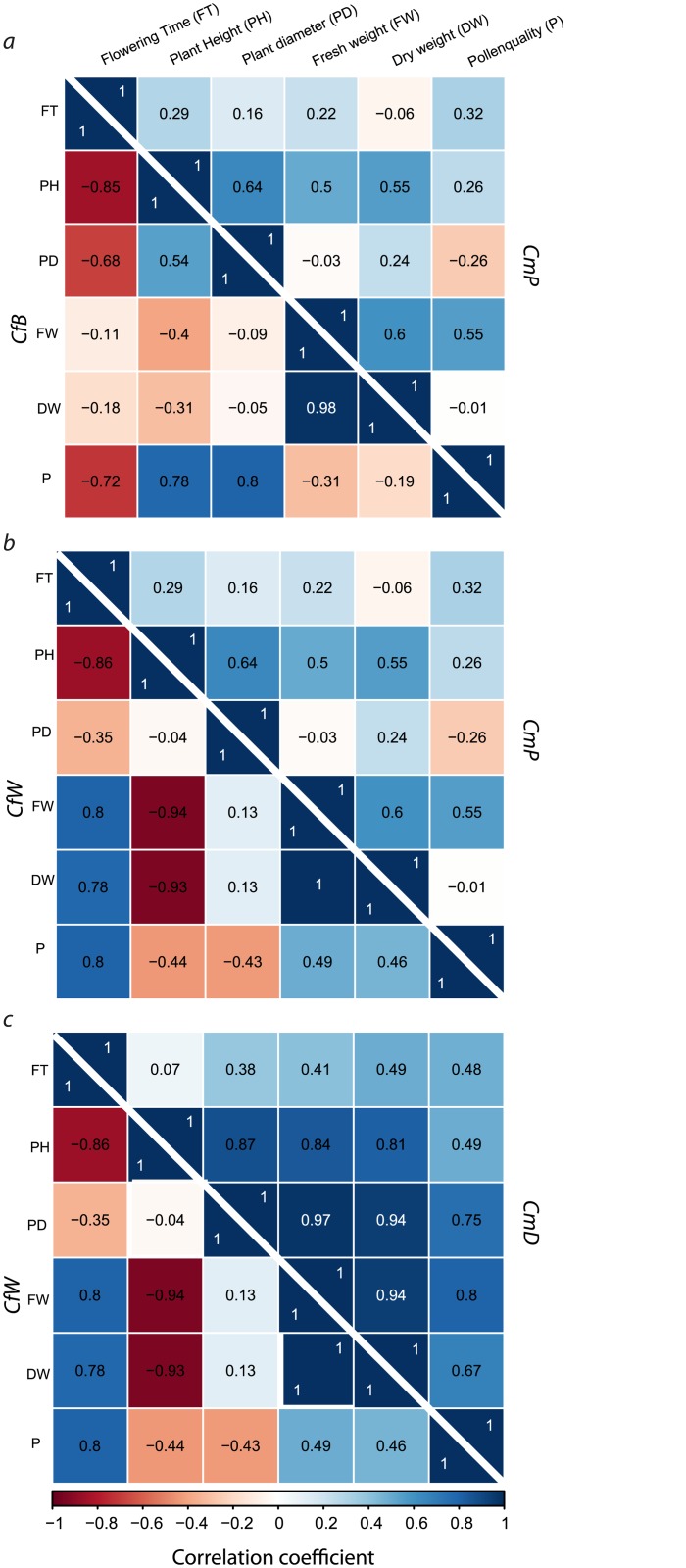
Correlation matrix presenting the correlation for each parental species separately. a: *Cf*B and *Cm*P; b: *Cf*W and *Cm*P; c: *Cf*W and *Cm*D. Correlation matrices are separated by the white line. Correlations of the parameters are expressed as Pearson correlation coefficients. The maximum positive correlation is given at a correlation coefficient of 1 (blue) and the maximum negative correlation at -1 (red). If no correlation exists between parameters, the correlation coefficient is 0 (white). P-values from correlation matrices are provided in S2 Table. Parental species and interspecific hybrids codes are provided in Table 1.

Cultivars from different species were selected for interspecific hybridisation with heterogeneous phenotypes. A correlation matrix was calculated to demonstrate the diversity of the breeding material (Fig 1, S2 Table). The parental species *Cf*B and *Cm*P were crossed in both directions to obtain interspecific hybrids. For *Cf*B, the correlations were mostly negative (Fig 1a, S2 Table). In contrast, the correlation pattern from *Cm*P exhibited predominatly positive correlations. The strongest negative correlation for this parental species was identified between PD and P with a correlation coefficient of -0.26.

The parental species *Cf*W and *Cm*P were crossed in one direction (Fig 1b, S2 Table). *Cf*W, selected as the maternal species, exhibited divergent correlation patterns in comparison to the paternal species of *Cm*P, by exhibiting strong positive as well as negative correlations of the parameters. A strong positive correlation between FW and FT of 0.8 existed, whereas the correlation between the parameters FW and PH was strongly negative (-0.94). This negative correlation represents a shorter shoot habitus showed increased FW (Fig 1b, S2 Table). Selected parameters from *Cm*P were only weakly positively correlated e.g. FT to FW with the value 0.22. The strongest correlation was identified between PH and PD (0.64).

*Cf*W and *Cm*D were crossed in one direction. *Cf*W was again used as the maternal species and showed both strong positive and negative correlations (Fig 1c, S2 Table). For *Cm*D, most of the parameters showed strong positive correlations. For this species, eight out of fifteen correlations were above 0.75. Additionally, no negative correlation could be identified.

In general, our results showed the heterogeneity of the breeding material, because of the different correlation patterns. No correlation matrix could be calculated for the parental species *Cc* and *Ci* because only one individual plant was analysed.

### Proof of Hybridity

**Fig 2:**
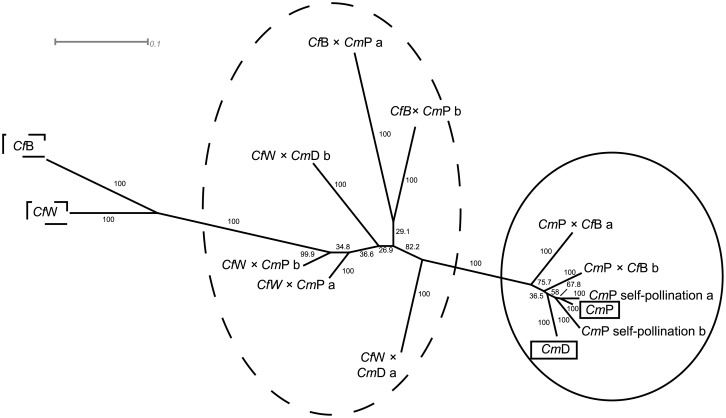
Neighbour-joining bootstrap consensus tree based on AFLP assay for parental *Campanula* species *Cm*P, *Cm*D, *Cf*W and *Cf*B and their interspecific hybrids. Numbers indicate bootstrap values for 1000 replicates. Parental species are labelled with squared boxes. All interspecific hybrids are surrounded by a dashed ellipse when *Cf* was used as the maternal species. In contrast, all interspecific hybrids are surrounded by a solid ellipse when *Cm* was selected as the maternal species. The scale represents relative genetic distance of 0.1. Parental species and interspecific hybrids codes are provided in Table 1.

A Neighbour-joining bootstrap consensus tree based on the AFLP assay was generated to demonstrate the genetic distance of the parental species. In general, the genetic distance between the *Cf* and *Cm* species was relatively high (Fig 2). When *Cf* was used as the maternal species, all interspecific hybrids were located between the parental species, indicating true hybridity. In contrast, the interspecific hybrids, where *Cm* was selected as the mother species, were genetically closer to both *Cm* species and to the self-pollinations of *Cm* E_1,2_, which was used as a control. The proximity indicated that interspecific hybrids of this cross direction might be derived from self-pollination. Interspecific hybrids of *Cf*B × *Cm*P G_1,2_ were closer related to each other in comparison to hybrids when *Cf*W was used as the mother species and crossed with *Cm*P or *Cm*D H_1,2_ and I_1,2_.

A Neighbour-joining bootstrap consensus tree was also calculated for the species *Cc* and *Ci* and the interspecific hybrids and showed a central position of the interspecific hybrid L_1,2_ between the parental species (S2 Fig)

The observations from the genetic distance (Fig 2) were confirmed by flow cytometry analyses (Table 3), which were used to identify interspecific hybrids. Relative DNA content was determined relative to the fluorescence intensity from the DAPI stained nuclei. For investigating the relative DNA content, 10,000 events were counted. The analyses resulted in 56833 ± 4836 counts for *Cm*P and 39615 ± 8025 counts for *Cf*B (Table 3). Interspecific hybrids of *Cf*B x *Cm*P G_1,2_ exhibited intermediate counts of 50.050 ± 270 and 52079 ± 409. The reciprocal crosses F_1,2_ had 58610 ± 3209 and 60615 ± 3651 counts, which were similar to the maternal species *Cm*P. Interspecific hybrids of each *Cf*W x *Cm*D’ and *Cm*P H_1,2_ and I_1,2_ also exhibited intermediate counts (Table 3). The interspecific cross with *Cc* as the maternal species had 32244 ± 4583 counts. With 34972 ± 4247 counts, the paternal species *Ci* had a similar number of stained nuclei. Interspecific crosses of *Cc* × *Ci* both L_1,2_ showed higher counts in comparison to both parental species of 39197 ± 4796 for L_1_ and 38315 ± 4669 for L_2_.

**Table 3:** Relative DNA content measured according to fluorescent from fluorescent, DAPI stained nuclei from parental species and interspecific hybrids.

	Maternal plant		Paternal plant				
Genotype code	species, cultivar		species, cultivar	Counts	Mean	SD	CV
A	*Cm*P			6333	56833	4836	8.5
D	*Cf*B			6817	39615	8025	20.3
F1	*Cm*P	×	*Cf*B	4947	58610	3209	8.4
F2	*Cm*P	×	*Cf*B	5133	60615	3651	8.2
G1	*Cf*B	×	*Cm*P	5858	50050	270	10.5
G2	*Cf*B	×	*Cm*P	5107	52079	409	9.9
C	*Cf*W			7946	3966	5351	13.5
B	*Cm*D			6093	52869	10478	19.8
H1	*Cf*W	×	*Cm*D	5358	51138	1654	9.3
H2	*Cf*W	×	*Cm*D	5771	54071	223	8.6
C	*Cf*W			7946	3966	5351	13.5
A	*Cm*P			6333	56833	4836	8.5
I1	*Cf*W	×	*Cm*P	5710	51807	541	10.0
I2	*Cf*W	×	*Cm*P	4973	53814	2322	9.1
E1	*Cm*P	×	*Cm*P	n.a.	n.a.	n.a.	n.a.
E2	*Cm*P	×	*Cm*P	n.a.	n.a.	n.a.	n.a.
J	*Ci*			8231	34972	4247	12.1
K	*Cc*			6156	32244	4583	14.2
L1	*Cc*	×	*Ci*	7327	39197	4796	12.3
L2	*Cc*	×	*Ci*	7578	38315	4669	12.2

DNA content for each species and interspecific hybrid based on counted stained nuclei, mean fluorescence intensity, standard deviation of the mean (SD), and coefficient of variation in percentage (CV) n.a. = not examined

The chromosome number of the parental species was determined by chromosome counting of root tips and was found to be within the values of 2n = 34 for *Cm*, 2n = 34 for *Cf* and 2n = 32 for *Ci*.

### Morphological Characterisation

**Fig 3:**
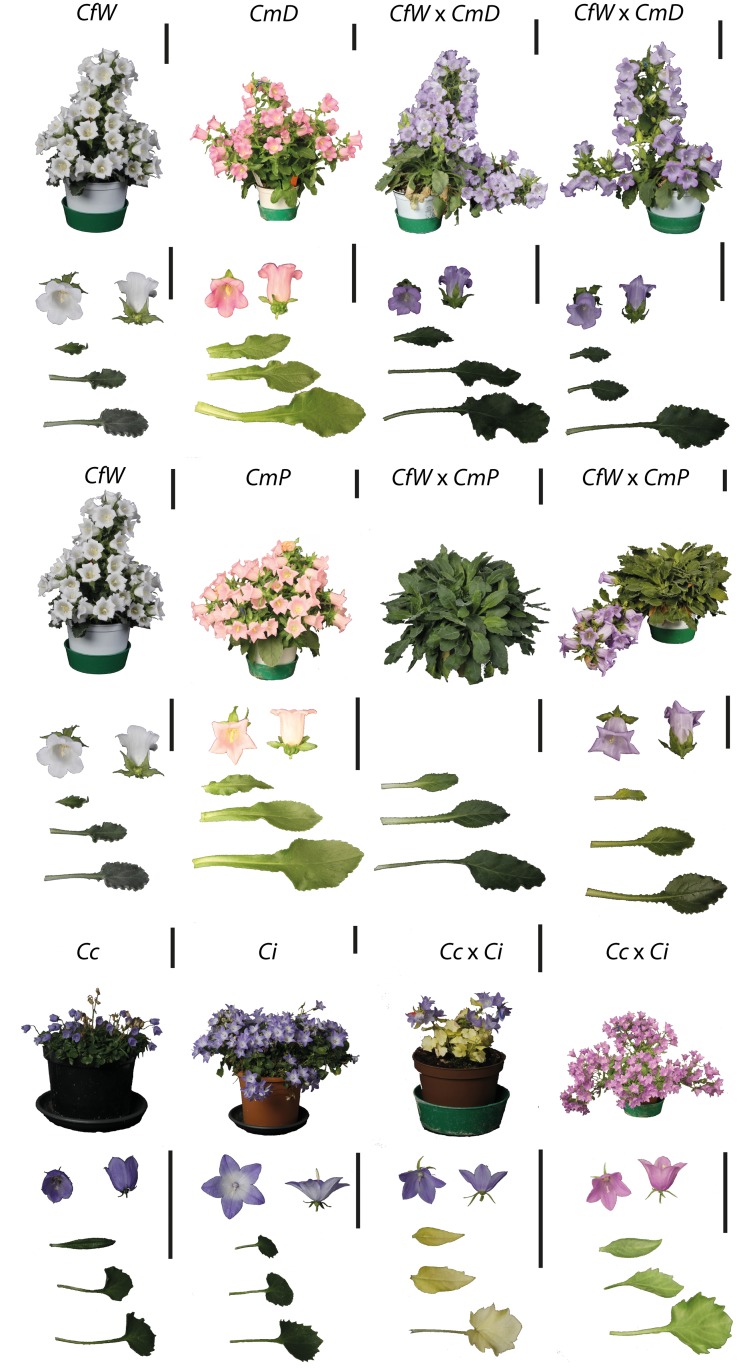
Morphology of shoot, flower and leaf shape from parental species and interspecific hybrids. a: *Cf*W C; b: *Cm*D B; c: *Cf*W x *Cm*D H_1_; d: *Cf*W x *Cm*D H_2;_ e: *Cf*W C; f: *Cm*P A; g: *Cf*W x *Cm*P I_1_; h: *Cf*W x *Cm*PI_2_; i: *Cc* K; j: *Ci* J; k: *Cc* x *Ci* L_1_; l: *Cc* x *Ci* L_2._ White scale bars represent 1 cm. Parental species and interspecific hybrids codes are provided in Table 1.

The parental species *Cm*D B and *Cf*W C differed in flower colour and flower shape. In addition, they showed differences in shoot habitus. When *Cf*W C was used as the maternal species, it had a solitary shoot, whereas *Cm*P A and *Cm*D D had a bushy shoot habitus (Fig 3). Interspecific hybrids of *Cf*W × *Cm*D H_1,2_ exhibited light violet flowers and a shoot morphology similar to the maternal plant species of *Cm*W.

When *Cf*W C was crossed with *Cm*P A, no plants of hybrid I_1_ induced flowers during the experiment. The flower induction was also inhibited in the I_2_ hybrid. Only one plant of hybrid I_2_ induced flowers (Fig 3). Interspecific hybrids G_1,2_ of *Cf*B D and *Cm*P A exhibited a similar purple flower colour, but differed from each other in petal structure and shoot habitus (S3 Fig). The petal tips from G_1_ were broader in comparison to the pointed petals of G_2_. G_1_ clearly exhibited a bushy shoot habitus, whereas G_2_ was solitary.

Parental species of interspecific hybrids *Cc* × *Ci* L_1,2_ differed morphologically in shoot, flower and leaf shape. *Cc* exhibited less shoot growth and smaller, more uniform coloured petals compared to *Ci*. Both parental species had intense green leaves. Interspecific hybrids of *Cc* × *Ci* L_1,2_ differed from each other in the CCI (Fig 3), flower number and shoot height. Hybrid L_1_ had yellowish leaves, which resulted in a low chlorophyll content index in comparison to the other green hybrid line L_2_. Additionally, the hybrid L_1_ had fewer open flowers per plant than hybrid L_2_.

**Fig 4:**
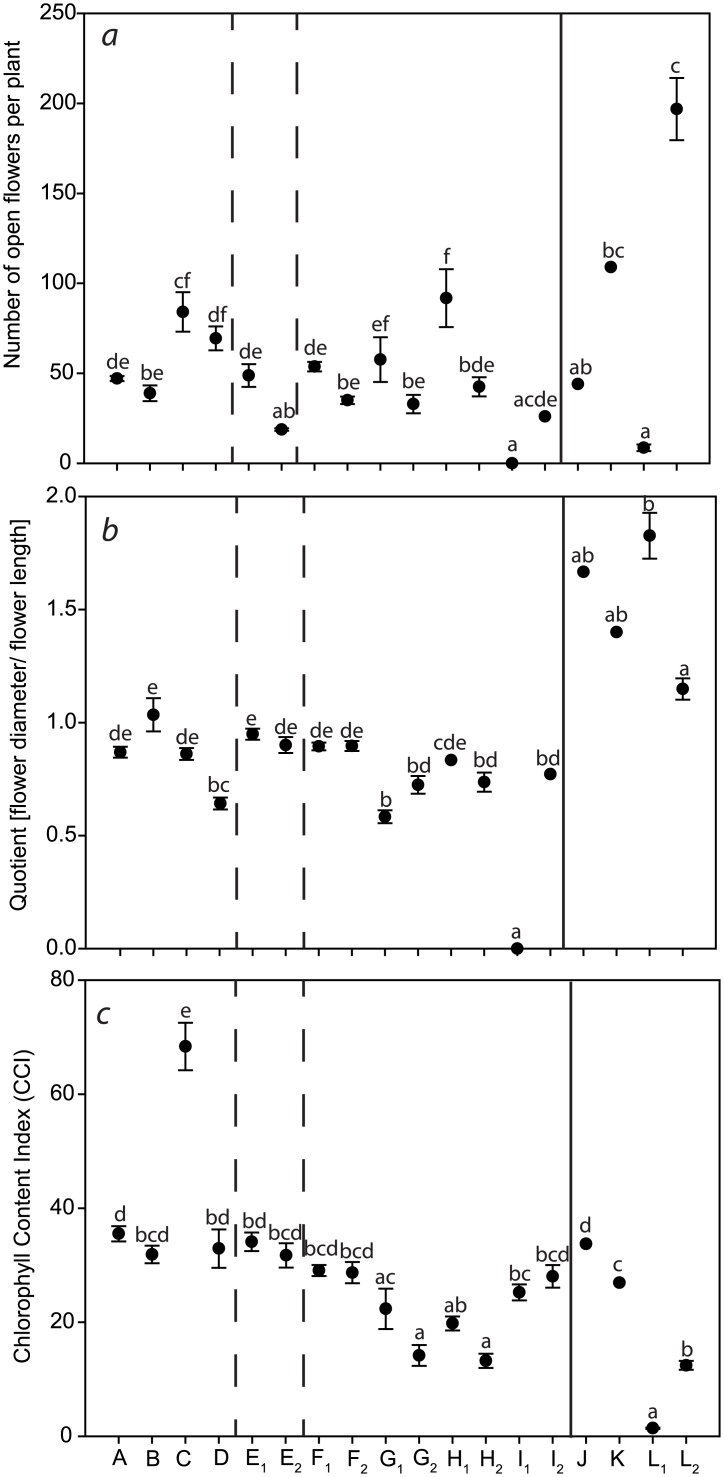
Biometrical analysis of parental species and interspecific hybrids. a: Number of open flowers per plant; b: Quotient of flower diameter and flower length; c: Chlorophyll Content Index. Parental species and interspecific hybrids codes are provided in Table 1.

Failure or delay of flower induction is a known indicator for incompatibility in wide hybridisation in *Arabidopsis* [10]. In addition, the NFP is an important criterion for the quality of ornamentals. To investigate the phenomenon of failed flower induction, the number of open flowers was counted. Furthermore, an ornamental plant with an increased number of flowers enhances the product value. Within the parental species both cultivars from *Cm*P and *Cm*D and *Cf*B and *Cf*W each had similar NFP (Fig 4a). Both self-pollinations of *Cm*P E_1,2_ differed significantly from each other in NFP. In general, most replicates of the two hybrid lines from all interspecific hybrids exhibited significant differences in the number of open flowers, e.g. *Cf*W x *Cm*D H_1_ had 92.0 ± 16.1 and H_2_ had 42.5 ± 5.3 open flowers (Fig 4a). No flowers were induced in the cross *Cf*W x *Cm*P I_1_ and only one plant induced flowers in the hybrid line I_2_. The NFP differed among the interspecific hybrids of *Cc* × *Ci*. Hybrid L_1_ had only 9.0 ± 1.9 open flowers, whereas the hybrid L_2_ exhibited 197.0 ± 17.3 open flowers (Fig 4a). The results demonstrated that a parental species significantly differs from the interspecific hybrids in general, but significant differences were also identified between the two related hybrid lines.

To describe the flower shape, a quotient of FD and FL was determined. This quotient demonstrated that the parental species did not exhibit a significantly different flower shape. Only *Cf*B D had a significantly elongated flower shape compared to the other parental species (Fig 4b). The self-pollinated *C*. *medium* hybrid lines E_1,2_ lacked a significantly different flower shape as hybrids of *Cm*P × *Cf*B *F*_*1*,*2*_. Flower shape of these crosses was round. Furthermore, they exhibited a higher flower quotient than the reciprocal cross. In the cross of *Cc* × *Ci* L_1,2_, both the parental species and the interspecific hybrids showed high quotients greater than 1, i.e. the flower shape was broad (Fig 4b). Nevertheless, both interspecific hybrid L_1,2_ were very contrary to each other as *Cc* × *Ci* L_2_ had the lowest quotient of 1.14 ± 0.05. In contrast, the hybrid line *Cc* × *Ci* L_1_ had the highest quotient of all used cultivars and interspecific hybrids with a value of 1.83 ± 0.10. Examinations of the flower shape show that all *Cm* cultivars, self-pollinations and interspecific hybrids, when *Cm* was used as the maternal species, have a wider campanulate flower shape. In the reciprocal cross, the flowers had an elongated shape. Interspecific hybrids of *Cc* × *Ci* as well as the parental species, exhibited a wider campanulate-shaped flower.

Lack of chlorophyll is an important indicator for incompatibilities between parental species in wide hybridisation. For this reason, the CCI was determined. Most parental species of *Cm* and *Cf* exhibited dark green leaves with a similar CCI ranging from approximately 32–36 (Fig 4c), with the exception of *Cf*W C, which had the darkest green leaves with the highest CCI (approx. 68). Both self-pollinated *C*. *medium* hybrids E_1,2_ also showed a similar CCI to the hybrids of *Cm*P × *Cf*B F_1,2_. Both interspecific hybrids of *Cf*B × *Cm*P G_1,2_ and *Cf*W × *Cm*D H_1,2_ had a significantly lower CCI than the parental species. Interspecific hybrids of *Cf*W × *Cm*P I_1,2_ exhibited a CCI similar to the parental species.

In the crosses of *Cc* × *Ci* L_1,2_ both interspecific hybrids had lower CCIs than the parental species, whereas plants from hybrid L_1_ clearly exhibited a lower CCI (1.4 ± 0.13 (Fig 4c)).

**Fig 5:**
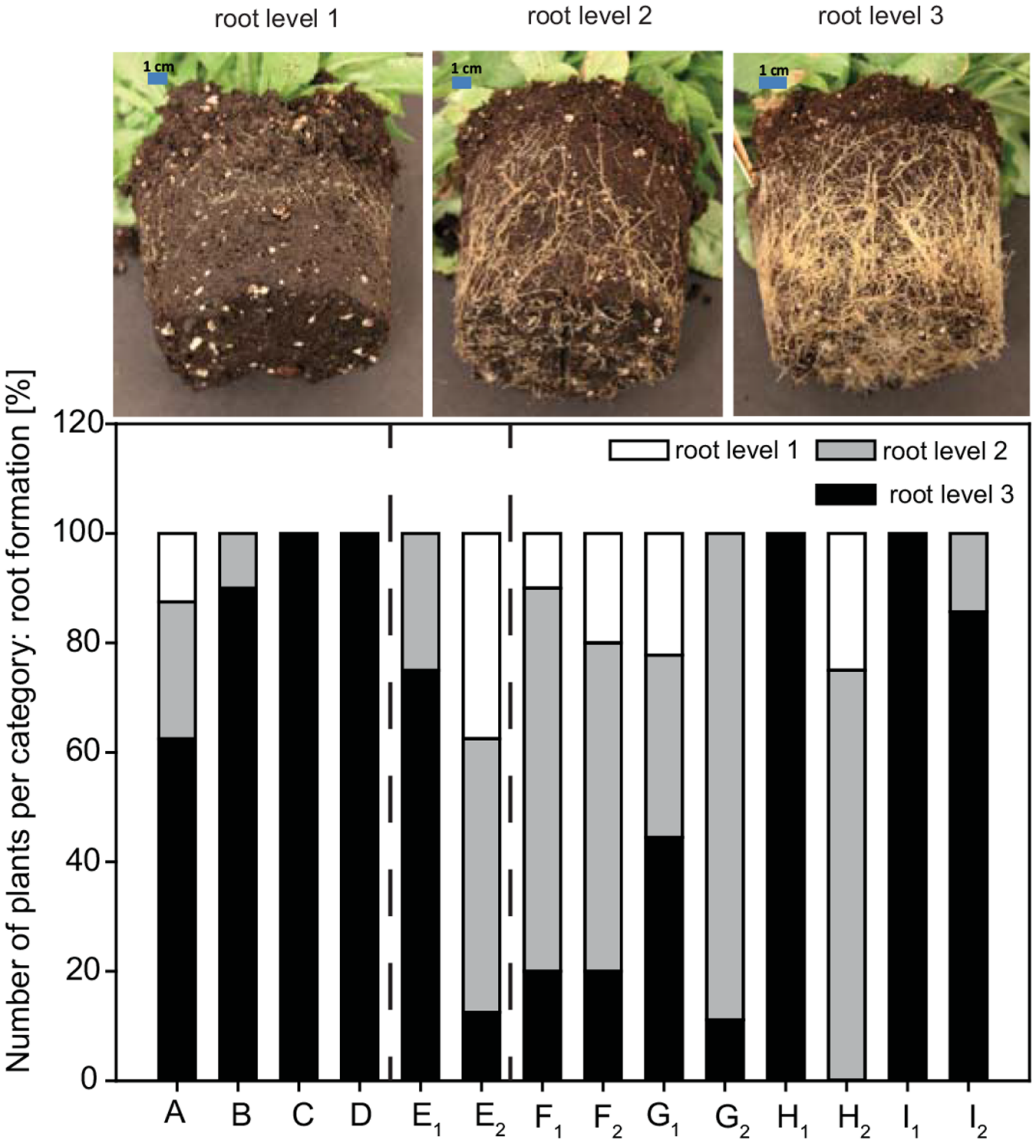
Root formation level (1–3) and percentage of plants per level. Parental species and interspecific hybrids codes are provided in Table 1.

RF is an important criterion for a potted plant. The investigation of the rooting levels illustrated that more than 60% of the plants from parental species exhibited an RF of level 3, which represented the highest RF (Fig 5). In general, approx. 66% of the interspecific hybrids exhibited a low number of plants with level 3 root formation. Only 20% of the interspecific hybrids *Cm*P × *Cf*B F_1,2_ had an RF level of 3, which demonstrated that most plants had a lower RF. In contrast, both interspecific hybrid I_1,2_ from *Cf*W and *Cm*P developed more roots, so that 85% and 100% of plants, respectively, achieved level 3 (Fig 5). Most interspecific hybrids had a reduced RF in comparison to the parental species.

**Fig 6:**
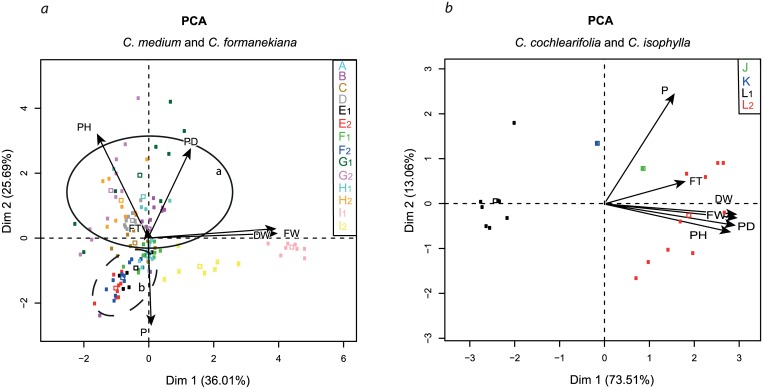
Principal component analysis (PCA) (Individuals factor map and variables factor map) displaying the variability of interspecific hybrids and parental species. PCA based on biometrical parameters: plant height (PH), plant diameter (PD), fresh weight (FW), dry weight (DW), pollen quality (P) and flowering time (FT). The variables factor map presents the amount of variance from each parameter on the total variance in the PCA. When the arrow is longer, then the amount from the total variance is larger. a: Values examined from parental species *Cm*P and *Cm*D and *Cf*B and *Cf*W (‘Blue’ and ‘White’). A squared box is the average of values for each parental species and interspecific hybrid. The solid ellipse includes all *Cf* plants and interspecific hybrids when *Cf* was the maternal species (cycle a). The dashed ellipse includes all *Cm* plants and interspecific hybrids when *Cm* was used as the maternal species (cycle b); b: Values originated from parental species *Cc*, *Ci* and interspecific hybrids. Parental species and interspecific hybrids codes are provided in Table 1.

The variables factor map, resulting from the PCA, showed the relation among the parameters from all parental species of *Cm* and *Cf* and the interspecific hybrids (Fig 6a). The variables factor map presents the amount of variance from each parameter on the total variance in the PCA. When the arrow is longer, the amount from the total variance is larger, i.e. the distribution of the individuals is wider. The first dimension explained approximately 36% of the total variation considering all parameters and described most of the variation of the parameters for both dry and fresh weight. Both parameters were described as strongly positively correlated by the orientation of the arrows. Dimension 2 explained approximately 26% of the total variation considering all parameters, presenting most of the variation of plant diameter and pollen quality. The variables factor map indicated a very low variation for the parameter flowering time. The results showed that nearly all parameters have a influence similar to the total variance, except flowering time, which had a lower influence compared to the other parameters (Fig 6a). In general, the PCA presents the morphological differences between the individual plants from A, B (*Cm*) and C, D (*Cf*) (Fig 6a, Table 1). Only individual plants of B (*Cm*D) overlapped in the phenotypic expression with individual plants from both A, B and C, D species. All hybrids from the crosses F_1,2_ (*Cm*P × *Cf*B) were located in the proximity of the parental species A (*Cm*P). The same observation was made for interspecific hybrids from the reciprocal cross G_1,2_. Here most of the interspecific hybrids were closely located to the C,D (*Cf*) species. Both interspecific crosses of H_1,2_ and I_1,2_ were separately located from both cycles a and b, which showed that both interspecific hybrids differed in the phenotype from all other *Cf* × *Cm* hybrids and both parental species.

The variables factor map represents the relationship among the parameters in the crosses between *Cc* and *Ci* (Fig 6b). The first dimension explained approximately 74% of the total variation considering all parameters and described most of the variation of the parameters PH, PD, and DW and FW. These parameters were described as strongly positively correlated. Dimension 2 explained approximately 13% of the total variation; none of the parameters were close to that dimension (Fig 6b). The results indicate that all parameters exhibited a similar influence to the total variation. Only FT showed a lower variance in comparison to the other parameters. The parameters of FT and P were both only weakly correlated with the other parameters.

In the PCA, the interspecific hybrids of *Cc* × *Ci* L_1,2_ were not clustered between the parental species *Cc K*, *Ci* J (Fig 6b). Both interspeciific hybrids of L_1_ and L_2_ had a different phenotype. The parental species of *Cc* K and *Ci* J are intermediately located between both interspecific hybrids L_1_ and L_2._ L_1_ expressed a phenotype more similar to the parental species *Cc* K, whereas L_2_ is more comparable to *Ci* J.

## Discussion

### Breeding Material

Breeding strategies in ornamental plants focus mainly on plant morphology. Flower morphology and leaf shape are especially important breeding goals. In the present study, the phenotypic variation was investigated by calculating a correlation matrix for each parental cultivar (Fig 1, S2 Table). The study showed that both *Cm* cultivars exhibited a similar phenotype with the strongest positive correlation between the selected morphological parameters. In contrast to *Cm* cultivars, *Cf* cultivars displayed another phenotype, presented by both strong positive and negative correlation of selected parameters. Our results confirmed that the selected breeding material is phenotypically heterogeneous and suitable for obtaining diverse offspring. Furthermore, similarity in chromosome numbers (*Cm*, *Cf*, *Cc* = 34 chromosomes, *Ci* = 32 chromosomes) and ploidy level (2n) should increase the likelihood of success in interspecific hybrids [25–28].

### Genetic Characterisation

For most of the interspecific hybrids, hybridity was confirmed by applying AFLP marker analysis (Fig 2). Application of AFLP assays to identify reliable hybrids is a suitable method, as reported in Bromeliaceae and Campanulaceae [20,39]. To demonstrate the genetic distance among parental cultivars and the interspecific hybrids, a Neighbour-joining bootstrap consensus tree was constructed, which identified three clear clusters (Fig 2). The first cluster represented the *Cf* cultivars, the second comprised the interspecific hybrids in the cross direction of *Cf* × *Cm*, and the third cluster included cultivars of *Cm*, self-pollination of *Cm*P and interspecific hybrids of the cross combination *Cm* × *Cf*. Both interspecific hybrid lines per cross combination in the cross direction of *Cf* × *Cm* exhibited low genetic distance from each other, except for the interspecific H_1_ and H_2_hybrids, which had a larger genetic distance (Fig 2). This is also indicated by the relative DNA content, where H_1_ had a lower amount than H_2_. Interspecific hybrids of *Cm*P × *Cf*B were identified as potential self-pollinations of *Cm*P. The phenotypic similarity of these interspecific hybrids with the maternal cultivar *Cm*P was confirmed by the very low genetic distance among them. Collectively, the results showed that AFLP markers could be used in future research for identifying interspecific hybrids in *Campanula*.

These results were verified through determination of the fluorescent nuclei related to the relative DNA content, which had a similar relative DNA content of 58610 ± 3208 and 60614 ± 3650 from interspecific hybrids of *Cm*P × *Cf*B and maternal cultivar *Cm*P with 56833 ± 4836 (Table 3). In the *Campanula* genus, self-pollination is often inhibited as reported for *C*. *dichotoma* [40]. To the authors’ knowledge, no reports exist describing autogamy in *Cm*. Our results indicate that *Cm*P is highly susceptible to self-pollination and should be carefully used as a maternal cultivar. Even though the flow cytometry confirmed the hybrid status of crosses between *Cm* and *Cf* cultivars, the method indicated difficulties in verifying interspecific hybrids of *Cc* × *Ci*. Here, the genome size was too similar; hence the flow cytometer could not differentiate the peaks, leading to incorrect values (Table 3). The problem of clearly identifying hybrids, when the parental cultivars have a low genetic distance, is a well-known issue. For *Centaurium*, the application of flow cytometry to identify hybrids was not possible, because the cultivars had very similar DNA content [41]. Nevertheless, with the conduction of AFLP marker analysis the identification of hybridity was successful. Both interspecific hybrid lines of *Cc* × *Ci* exhibited equal genetic distance to the parental cultivars (S2 Fig). Collectively, our results proved the suitability of the AFLP marker-based analysis to confirm interspecific hybrids in *Campanula* and suggest a critical use of the flow cytometry method, because similar genome sizes could not be determined.

### Morphological Characterisation

Selected interspecific hybrids exhibited interesting traits, which differed from their parental cultivars. Both interspecific hybrid lines H_1,2_ of *Cf*W × *Cm*D had similar shoot habitus, but clearly differed in flower colour in comparison to the parental cultivars (Fig 3). The genetic distance of both interspecific hybrid lines H_1,2_ from the parental cultivars was found to be similar, but the phenotype was strongly determined by the maternal cultivar *Cf*W.

For the cross combination of *Cf*W × *Cm*P, *Cf*W was also used as the maternal cultivar, whereas *Cm*D was chosen as the paternal cultivar. Failed flower induction (only one plant was flowering) was observed (Fig 3). One explanation for this could be that this cross combination needed a longer period of vernalisation for flower induction. Furthermore, failed flower induction could be caused by incompatibilities, as reported for interspecific hybrids of *Arabidopsis* [10]. NFP is an important ornamental parameter; therefore, further investigations in *Campanula* are needed to determine the reason for this incompatibility.

The interspecific hybrids L_1,2_ from the cross combination of *Cc* × *Ci* had similar phenotypes, but differed in the shoot height and flower number. Especially the lack of chlorophyll in hybrid L_1_ could be an indication of incompatibilities between the parental cultivars as observed in interspecific hybrids of *Lonicera* (*L*. *caerulea* × *L*. *gracilipes*) [18]. Studies on nuclear-cytoplasmic incompatibility in *Pea* demonstrated that insufficient chlorophyll can be a result of unusual biparental plastome DNA inheritance [42]. The normal inheritance of plastid DNA through maternal, parental or bilateral directions in generative propagation cannot explain the phenomenon that only L_1_ is lacking chlorophyll. Interestingly, this observation showed that both related hybrids lines can exhibit traits differently.

The different NFP, flower size and CCI revealed differences among the cross combinations as well as between the two hybrid lines for each cross combination. Most studies on interspecific hybrids are lacking the comparison of two related hybrid lines. These comparisons are important to determine the stability of inherited traits. Only the investigation of two or more hybrids will demonstrate the variation within the cross combinations. The phenotypic traits of both NFP and the quotient of FD and FL for the interspecific hybrids of *Cm* and *Cf* showed an intermediate phenotype in comparison to the parental cultivars (Fig 4a and 4b). Additionally, differences between the two hybrid lines were explored. Further research would be needed to investigate the segregation of the traits. Interestingly, interspecific hybrids of *Cc* × *Ci* differed from both parental cultivars e.g. in the flower quotient (Fig 4b).

Investigations of the CCI in interspecific hybrids of *Cm* and *Cf* demonstrated that most interspecific hybrids exhibited no significantly different CC in comparison with at least one parental cultivar (Fig 3c). Only hybrids G_2_ and H_2_ had significantly lower CCIs (Fig 4c).

Both interspecific hybrids of *Cc* × *Ci* L_1,2_ showed significantly lower CCI in comparison to both parental cultivars. L_1_ could be classified as an albino plant, due to a very low CCI. Our study indicated higher incompatibilities between these crossed species in comparison to the crosses between *Cm* and *Cf*. Molecular studies on albino wheat plants showed a lack of plastid ribosomes, altered transcription and translations pattern in comparison to green plants [43]. Moreover, embryogenesis examination of barley microspores detected genes in relation to albinism [44].

Most of the interspecific hybrids displayed reduced RF (Fig 5), a phenomenon which has not been described for other hybrids. Presumably, RF was not the focus when describing new interspecific hybrids. Our results showed that interspecific hybridsation in the selected *Campanula* cultivars mostly resulted in plants with reduced RF ability. However, this study indicates that RF is a trait that should be explored when evaluating new hybrids.

Conduction of PCA to explore the relationship of traits is commonly applied in field crop breeding to identify correlated traits [45, 46]. Correlated traits will influence each other when one trait is the focus of a breeding strategy. In this study it was the aim to conduct PCA to investigate the potential use for ornamental breeding. Correlation among selected traits were examined to determine the phenotypic relationship of interspecific hybrids according to the selected traits. The PCA revealed distributions of the phenotypes which differed from the intespecific hybrids. Most *Cm* cultivars were clustering closely together with the interspecific hybrids of *Cm*P × *CfB* in cycle b (Fig 6a). In contrast, most individuals of *Cf*, cultivars and interspecific hybrids of *Cf* × *Cm* were widely separated from each other within cycle a. PCA results suggest a greater influence on the phenotypic formation from the maternal plant species, because most of the interspecific hybrids exhibted a characteristic trait similar to the maternal cultivar (Fig 6a).

Both interspecific hybrids of *Cc* × *Ci* were completely different in their traits in comparison to each other. Each of them was similar to one of the parental cultivars. This observation was unexpected, because both hybrids are sibling lines in the same cross direction. It was estimated that both hybrid lines L_1,2_ showed a higher similarity to the maternal cultivar *Cc*, as observed in most of the interspecific hybrids of *Cf* and *Cm*. Additionally, the PCA can be recommended as a method of comparing phenotypes based on many biometrical parameters to analyse the phenotypic distribution.

In general, the presented results clearly demonstrate the usefulness of genetic analyses combined with phenotyping methods to evaluate newly combined traits and to characterise the novel interspecific hybrids.

## Conclusion

Collectively, genetic distances between the parental cultivars were presented; the hybridity status of obtained hybrids was proven in crosses of *Cf* × *Cm* cultivars and *Cc* × *Ci*. Interspecific hybrids of the reciprocal cross *Cm* × *Cf* were identified as self-pollinations. The phenotypic variation was determined by biometrical data in the morphological analysis. Our results prove the usefulness of the AFLP DNA marker system to verify interspecific hybrids for the selected *Campanula* cultivars. The comprehensive study of both phenotypic and genotypic data makes it possible to optimise breeding strategies in *Campanula* and to evaluate hybrid performance.

## Supporting Information

S1 Fig Allele diversity of AFLP primer. (EPS)

S2 Fig Neighbour-joining bootstrap consensus tree based on AFLP assay for parental *Campanula* species *Cc*, *Ci* and their interspecific hybrids. (EPS)

S3 Fig Morphology of shoot, flower and leaf shape from parental species *Cm*P, *Cf*B and interspecific hybrids. (EPS)

S1 Table AFLP primers, number of polymorphic alleles, average PIC and average allele diversity. (DOCX)

S2 Table P-values for correlation matrix (Fig 1), with species abbreviation provided in Table 1. (DOCX)
