# Supplementary material for: Phenotypic and Genotypic Analysis of Newly Obtained Interspecific Hybrids in the Campanula Genus
Source: PLoS One. 2015 Sep 9;10(9):e0137537. doi: 10.1371/journal.pone.0137537 (PMC4564236; doi:10.1371/journal.pone.0137537)
Supplement: S1 Table — (DOCX) [file pone.0137537.s004.docx]

|  | ***Campanula* species** | | | |
| --- | --- | --- | --- | --- |
|  | *Cf*W | *Cf*B | *Cm*P | *Cm*D |
| Parameter | p- value | p- value | p- value | p- value |
| FT-PH | 0.41 | 0.02 | 0.06 | 0.84 |
| FT-PD | 0.65 | 0.09 | 0.56 | 0.28 |
| FT-FW | 0.55 | 0.81 | 0.11 | 0.24 |
| FT-DW | 0.86 | 0.70 | 0.12 | 0.15 |
| FT-P | 0.36 | 0.07 | 0.10 | 0.16 |
| PH-PD | 0.05 | 0.21 | 0.95 | 0.00 |
| PH-FW | 0.14 | 0.38 | 0.02 | 0.00 |
| PH-DW | 0.10 | 0.49 | 0.02 | 0.00 |
| PH-P | 0.47 | 0.04 | 0.46 | 0.15 |
| D-FW | 0.92 | 0.84 | 0.83 | 0.00 |
| D-DW | 0.50 | 0.91 | 0.84 | 0.00 |
| D-P | 0.47 | 0.03 | 0.47 | 0.01 |
| FW-DW | 0.06 | 0.00 | 0.00 | 0.00 |
| FW-P | 0.10 | 0.50 | 0.40 | 0.01 |
| DW-P | 0.99 | 0.68 | 0.43 | 0.04 |

**S1 Table. P-values for correlation matrix ( Fig 1), with species abbreviation provided in Table 1.**
