# Supplementary material for: Phenotypic and Genotypic Analysis of Newly Obtained Interspecific Hybrids in the Campanula Genus
Source: PLoS One. 2015 Sep 9;10(9):e0137537. doi: 10.1371/journal.pone.0137537 (PMC4564236; doi:10.1371/journal.pone.0137537)
Supplement: S2 Table — (DOCX) [file pone.0137537.s005.docx]

**S2 Table. AFLP primers, number of polymorphic alleles, average PIC and average allele diversity.**

| **Marker name** | **Sequence** | **Number of polymorphic alleles (%)** | **Average PIC*** | **Average allele diversity** |
| --- | --- | --- | --- | --- |
| MseI- 50 | 5'-GATGAGTCCTGAGTAACAT-3' | 58 (72) | 0.31 | 0.35 |
| Pstl - 16 | 5'-GACTGCGTACATGCAGCC-3' |  |  |  |
|  |  |  |  |  |
| MseI- 62 | 5'-GATGAGTCCTGAGTAACTT-3' | 88 (64) | 0.30 | 0.33 |
| Pstl - 20 | 5'-GACTGCGTACATGCAGGC-3' |  |  |  |
|  |  |  |  |  |
| MseI- 47 | 5'-GATGAGTCCTGAGTAACAA-3' | 77 (73) | 0.33 | 0.35 |
| Pstl - 35 | 5'-GACTGCGTACATGCAGACA-3' |  |  |  |
|  |  |  |  |  |
| MseI- 49 | 5'-GATGAGTCCTGAGTAACAG-3' | 86 (66) | 0.33 | 0.47 |
| Pstl - 11 | 5'-GACTGCGTACATGCAGAA-3' |  |  |  |

*PIC= Polymorphism information content
